# Quantifying camouflage: how to predict detectability from appearance

**DOI:** 10.1186/s12862-016-0854-2

**Published:** 2017-01-06

**Authors:** Jolyon Troscianko, John Skelhorn, Martin Stevens

**Affiliations:** 1 University of Exeter, Centre for Ecology and Conservation, College of Life & Environmental Sciences, Penryn Campus, Penryn, Cornwall TR10 9FE UK; 2 Centre for Behaviour and Evolution, Institute of Neuroscience, Newcastle University, Henry Wellcome Building, Framlington Place, Newcastle upon Tyne, NE2 4HH UK

**Keywords:** Animal coloration, Background matching, Camouflage, Crypsis, Disruptive coloration, Image processing, Pattern analysis, Predation, Signalling, Vision

## Abstract

**Background:**

Quantifying the conspicuousness of objects against particular backgrounds is key to understanding the evolution and adaptive value of animal coloration, and in designing effective camouflage. Quantifying detectability can reveal how colour patterns affect survival, how animals’ appearances influence habitat preferences, and how receiver visual systems work. Advances in calibrated digital imaging are enabling the capture of objective visual information, but it remains unclear which methods are best for measuring detectability. Numerous descriptions and models of appearance have been used to infer the detectability of animals, but these models are rarely empirically validated or directly compared to one another. We compared the performance of human ‘predators’ to a bank of contemporary methods for quantifying the appearance of camouflaged prey. Background matching was assessed using several established methods, including sophisticated feature-based pattern analysis, granularity approaches and a range of luminance and contrast difference measures. Disruptive coloration is a further camouflage strategy where high contrast patterns disrupt they prey’s tell-tale outline, making it more difficult to detect. Disruptive camouflage has been studied intensely over the past decade, yet defining and measuring it have proven far more problematic. We assessed how well existing disruptive coloration measures predicted capture times. Additionally, we developed a new method for measuring edge disruption based on an understanding of sensory processing and the way in which false edges are thought to interfere with animal outlines.

**Results:**

Our novel measure of disruptive coloration was the best predictor of capture times overall, highlighting the importance of false edges in concealment over and above pattern or luminance matching.

**Conclusions:**

The efficacy of our new method for measuring disruptive camouflage together with its biological plausibility and computational efficiency represents a substantial advance in our understanding of the measurement, mechanism and definition of disruptive camouflage. Our study also provides the first test of the efficacy of many established methods for quantifying how conspicuous animals are against particular backgrounds. The validation of these methods opens up new lines of investigation surrounding the form and function of different types of camouflage, and may apply more broadly to the evolution of any visual signal.

**Electronic supplementary material:**

The online version of this article (doi:10.1186/s12862-016-0854-2) contains supplementary material, which is available to authorized users.

## Background

Animal coloration often plays a major role in survival and reproduction. Consequently, various forms of coloration—from camouflage to mimicry—have long been used as key examples of evolution by natural selection, and have provided an important test-bed for evolutionary thinking [1–3]. More recently, studies of colour patterns have made substantial progress in understanding the types of defensive coloration that exist, and the mechanisms that make them effective [4–6]. Progress has perhaps been most marked in the study of concealment, with camouflage one of the most common anti-predator strategies in nature [7, 8]. However, predicting an animal’s detectability from its visual appearance remains challenging. This is an important problem because quantifying how well an animal avoids detection against a particular background is key to investigating a wide range of evolutionary and ecological hypotheses surrounding animal signalling and survival. Moreover, it will also allow us to design effective camouflage patterns for human use: to disguise items as diverse as telephone masts, cameras, buildings and military equipment and personnel. Indeed, rigorously quantifying colour patterns in general has been a topic of considerable recent interest due to both the potential human applications and the unique opportunity to revolutionise our understanding of animal signalling [9–11]. Regarding camouflage, identifying and assessing the effectiveness of a wild animal’s camouflage strategy is essential for understanding predator–prey interactions in any system with visually guided predators. Many conspicuous displays will also be influenced by evolutionary pressure for greater camouflage, for example, displays that are aposematic at close quarters can be cryptic from further away [12], and conversely, an understanding of how camouflage is achieved can illuminate the mechanisms that make conspicuous coloration effective [13]. Quantifying an animal’s appearance relative to its background is therefore essential for investigating sexual, aposematic and camouflaged displays in a diverse range of fields.

A number of different types of camouflage have been identified, based on how they hinder detection. The most ubiquitous camouflage strategy is probably background matching, where animals match the general colour, brightness and patterns of their backgrounds [8, 14]. However, one of the key features thought to facilitate detection and/or recognition is the overall outline of an object. Here, high-contrast markings intersecting an animal’s outline may be used to ‘disrupt’ the viewer’s ability to discern or detect it [4, 7, 15], a strategy called disruptive coloration. The most direct way to determine an animal’s camouflage, and how effective it is, uses often lengthy behavioural tests or survival experiments that are difficult to undertake in the wild [16]. Considerable effort has therefore pursued computer models that can quantify how well any two visual samples match according to different processing or camouflage theories [17–22]. However, these camouflage models have rarely, if ever, been directly compared under controlled conditions, nor using data on observer success in finding hidden objects. This lack of model validation means that researchers rarely know which methods they should adopt when investigating an animal’s appearance. Furthermore, models that quantify visual signals and their match (or contrast) with the background have the potential to greatly inform us regarding the mechanisms through which colour patterns work, and how they should be optimised for maximal success (or indeed, traded-off with other competing functions). If models that are based on specific or generalised features of visual processing fit with behavioural data, this can illuminate the possible mechanisms through which colour patterns are made effective [19], and even how changes to them might improve the adaptive value of the defence. Where the models are inspired by known or likely neural architecture this can even reveal likely avenues for research into the underlying structures performing the visual processing.

**Fig. 1:**
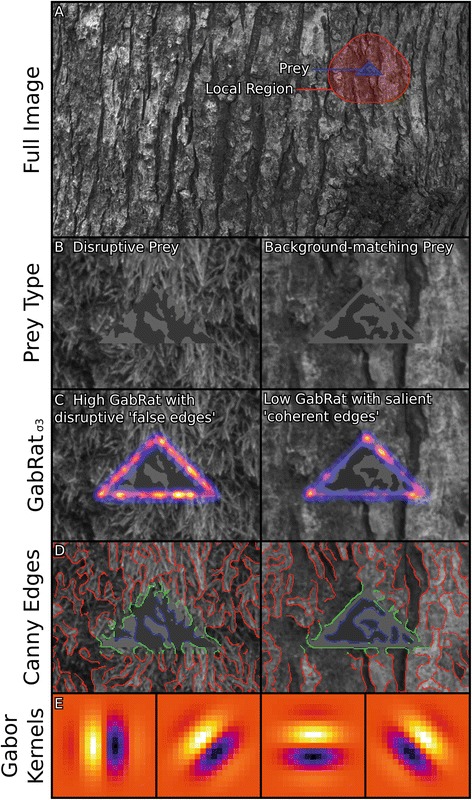
Examples of prey and edge disruption measurements. (**a**) Sample prey highlighted in *blue* against its background image. The ‘local’ region within a radius of one body-length is highlighted in *red*. (**b**) Examples of prey generated with the disruptive algorithm (*left*) and background-matching algorithm (*right*). These prey were chosen as their GabRat values were near the upper and lower end of the distribution (see below). (**c**) Illustration of the GabRat measurement. Red and yellow false colours indicate the perceived edges run orthogonal to the prey’s outline (making disruptive ‘false edges’), *blue* false colours indicate the perceived edges are parallel to the prey’s outline (making ‘coherent edges’). GabRat values are only measured on outline-pixels, so these values have been smoothed with a Gaussian filter (σ = 3) to illustrate the approximate field of influence. The prey on the *left* has a high GabRat value of 0.40, while the prey on the *right* has a low GabRat value (0.20). (**d**) Canny edges are highlighted in the images. Edges inside the prey are highlighted in *blue*, edges in the prey’s outline region are *green*, and edges outside the prey are *red*. The VisRat and DisRat disruption metrics are formed from the ratios of these edges. (**e**) Gabor filter kernels (sigma = 3), shown in false colour at the different angles measured

Here we set out to address the above issues by pitting numerous contemporary models of camouflage directly against one another, using human observers to compare models to detection times. We tested seven classes of model that have been previously used for investigating background matching and disruptive coloration hypotheses. These models compare prey to their backgrounds according to three different criteria: i) luminance matching, ii) pattern matching and iii) disruptive coloration. Luminance (i.e. perceived lightness) matching is the simplest form of camouflage to measure, for example calculating the average difference in luminance between prey and their backgrounds, which is thought to be important in explaining survival in peppered moths against mismatching backgrounds [23]. Contrast differences measure how similar the variation in luminance in the prey is to that of the prey’s background. This has been found to be important in the survival of wild ground-nesting bird clutches [16]. Pattern matching is another important aspect of background-matching camouflage that has recently been found to predict the survival of nightjar nests [16]. Pattern matching has been measured using a number of different methods that vary in their biological plausibility and complexity. These methods generally separate images into a series of spatial scales (e.g. using fast Fourier transforms, or Gabor filters), then compare the information at these different scales between the prey and background. Other methods search for shapes or features found in the prey that are similar to those in their backgrounds [22, 24]. For an overview see Table 1 and Additional file 1. The final type of camouflage measured was disruptive coloration, where contrasting markings break up an object’s outline and create false edges [7, 8, 15]. This camouflage strategy has received considerable investigation in the last decade, and has been shown to be highly successful in numerous contexts, including where stimuli have various contrast and pattern features manipulated [4, 25–30]. In spite of the clear protective benefits of artificial disruptive edges, it has proven far more difficult to measure how disruptive real prey are against their backgrounds [19, 31]. Recent edge disruption measures have quantified how many edges are present in the animal’s outline [32], or have used the Canny edge detector to measure the number of perceived edges in the prey’s outline relative to its surroundings [21], or the number of outline edges relative to those in the object’s centre [33]. However, these measures do not take into account the direction of the perceived edges, so cannot distinguish ‘false edges’ (that run at right angles to the prey’s outline and should be maximally disruptive [8, 31]) from ‘coherent edges’ that match the angle of the animal’s outline, making the prey’s tell-tale shape easier to detect [34]. We therefore developed a novel edge disruption metric called ‘GabRat’ that uses biologically inspired and angle-sensitive Gabor filters to measure the ratio of false edges to coherent edges around a target’s outline (see Fig. 1e) [35–37]. A high ratio of false edges to coherent edges should be more disruptive, making prey more difficult to detect. Background complexity is also known to influence detection times [38]. For example, artificial moths were more difficult to find for humans and birds alike when the surrounding bark had higher levels of luminance contrast and edge orientation changes [24]. However, in this study we aimed to focus on metrics that investigate the interaction between target and background, rather than assess the general properties of backgrounds that affect concealment.

**Table 1:** Descriptions of the methods used to measure prey conspicuousness

Camouflage Category	Variable Name	Filtering Method	Basic Description
Edge Disruption	GabRat	Gabor Filter	Average ratio of ‘false edges’ (edges at right angles to the prey outline) to ‘salient edges’ (edges parallel with the prey outline). See Additional file 1
	VisRat	Canny Edge Detector	Proportion of Canny edge pixels in the prey’s outline region [21]
	DisRat	Canny Edge Detector	Proportion of Canny edge pixels in the prey’s outline region [34]
	Mean Edge-region Canny Edges	Canny Edge Detector	Proportion of Canny edge pixels in the prey’s outline region.
	Edge-intersecting cluster count	None	Count of the number of changes in the pattern around the prey’s outline [33]
Pattern/Object detection	SIFT	Difference-of-Gaussians, Hough Transform	Uses Hough transform to find features in the prey, then counts how many similar features are found in the background [19, 22]
	HMAX	Gabor Filter	Breaks down a bank of Gabor Filter outputs into layers that describe patterns with some invariance to scale and orientation [20]
Pattern	PatternDiff	Fourier Bandpass	Sums the absolute difference between the prey’s pattern statistics [42]
	Euclidean Pattern Distance	Fourier Bandpass	Euclidean distance between normalised descriptive pattern statistics [42]
Luminance	Mean Backgorund Luminance	Luminance	Mean luminance
	Mean Luminance Difference	Luminance	Absolute difference between mean prey and mean background luminance
	LuminanceDiff	Luminance	Sum of absolute luminance histogram bins [16]
	Contrast Difference	Luminance	Absolute difference of contrast between prey and background, where contrast is the standard deviation in luminance levels

We tested how the above camouflage models predicted the performance of human ‘predators’ searching for camouflaged stimuli against natural background images on a touch screen monitor. Each prey was unique, generated from its background using methods that maximised or minimised the prey’s level of edge disruption, with prey also varying in their level of background matching. We used tree-bark backgrounds as these are biologically relevant backgrounds for a wide range of camouflaged animals, and they exhibit a degree of background heterogeneity in contrast and spatial features. Artificial prey and tree-bark backgrounds such as these have been used extensively for testing camouflage theories because they capture the essence of camouflage patterns effectively without the need to find and take calibrated images of large numbers of camouflaged prey [4, 27, 28, 32]. These studies have also demonstrated that human and non-human visual systems respond similarly to these camouflage stimuli. We calculated the preys’ camouflage with a battery of different models to determine which best predicted capture times. Each prey’s level of camouflage was measured between their entire background image, or with the local region within one body length to investigate whether camouflage is best predicted by local or global background matching. In addition we tested for interactions between the most effective luminance-, pattern- and disruption-based camouflage metrics to determine whether extreme luminance differences render pattern and disruption strategies ineffective. Finally, we discuss the effectiveness of the most commonly used models for assessing an animal’s camouflage and what our findings reveal about the mechanisms underlying animal camouflage.

## Results

**Fig. 2:**
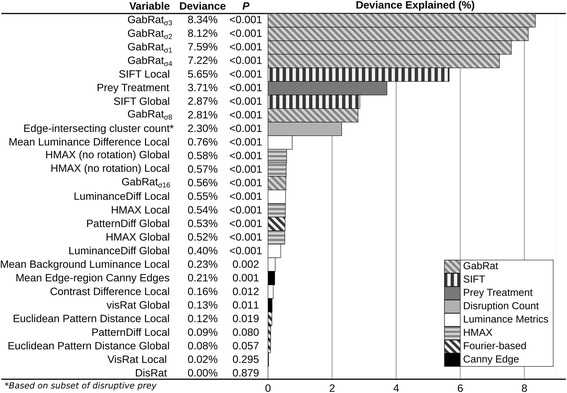
Capture time prediction accuracy. The predictive performance of camouflage metrics tested in this study ranked from best to worst. All camouflage metrics were continuous variables using one degree of freedom in each model with the exception of treatment type, which was categorical, consuming two degrees of freedom. Note that DisRat and VisRat performed better when fitted with a polynomial

There were substantial differences between the abilities of different camouflage metrics to predict capture times, see Fig. 2 for full results. Camouflage experiments such as this are expected to entail a very high levels of residual variation in capture times due to the interaction between the location of the prey, the viewers’ eye movements [32], and the heterogeneous nature of the backgrounds. For example, prey that appeared in the centre of the screen were captured sooner than those nearer the edges, explaining 8.75% of model deviance in the best model, while the random effects of participant ID and background image ID explained 3.16 and 1.68% of variance respectively. The best predictor of capture times was GabRat_σ3_, which measured edge disruption and explained 8.34% of model deviance (*p* < 0.001). As an illustration of effect size, prey in the upper 10% of GabRat_σ3_ values took on average 2.23 times longer to catch than those in the lower 10% of GabRat_σ3_ values (4.447 s as opposed to 1.998 s). This was followed by the Local SIFT model (measuring pattern/feature similarities, explaining 5.65% deviance, *p* < 0.001), and together with other GabRat sigma values (which specify the size of the filter), these were the only metrics that performed better than the prey treatment (i.e. whether it was generated using the background matching or disruptive algorithm, which explained 3.71% deviance, *p* < 0.001). The worst metric at predicting capture times was the Canny edge disruption measurement DisRat, explaining less than 0.01% of model deviance (*p* = 0.879), although this was likely due to its non-linear nature, see below.

The full model comparing the best edge disruption, pattern and luminance predictors contained GabRat_σ3_, Local SIFT difference and Mean Local Luminance Difference metrics. Following AIC simplification the model retained an interaction between GabRat_σ3_ and SIFT local that explained 0.21% deviance, with the main effect of GabRat_σ3_ explaining the majority of deviance (8.18%) and SIFT local with (3.05%) all terms were significant (*p* < 0.001). The global comparisons model based on bandpass descriptive statistics performed comparatively well, explaining 1.87% of deviance when summed across all model terms. This model contained four two-way interactions that retained all five descriptive variables (full model output is available in Additional file 1). The local comparisons model using bandpass descriptive statistics performed less well, retaining just Dominant Spatial Frequency Difference as a predictor that explained 0.42% of deviance). While background complexity measured independently of the prey was not the focus of this study, a number of metrics effectively include this information, such as the Mean edge-region Canny edges (deviance = 0.21%, *p* = 0.001), and Mean Local Bandpass Energy (deviance = 0.19%, *p* = 0.002).

Gabor-based pattern-matching metrics did not vary consistently between local and global difference predictors. The bandpass-based pattern matching metrics performed better when comparing the prey to their global region than their local region with the exception of the Euclidean Pattern Distance, which performed better locally. In contrast, luminance metrics all performed better when considering the local rather than global regions. However this is perhaps to be expected given the way the background images were normalised, and the way prey were generated from their backgrounds. Nevertheless, the Global PatternDiff metric performed substantially better than the Global Mean Luminance Difference, which as predicted is non-significant (deviance = 0.04%, *p* = 0.143).

Given the striking difference in performance of DisRat and GabRat metrics we tested how well each of them predicted prey treatment. As predicted, disruptive prey had a significantly lower DisRat and higher GabRat_σ3_ than background-matching prey (linear model; DisRat: F_1, 3817_ = 1413, *p* < 0.001; GabRat_σ3_: F_1, 3817_ = 708.2, *p* < 0.001), demonstrating that both were able to predict treatment type. When fitted with a quadratic, VisRat local and DisRat both fitted capture times significantly better (based on model comparison, *p* < 0.005), increasing the deviance explained by these variables to 0.439 and 0.558% respectively. The optimal VisRat local ratio was equal to 0.951, while the optimum DisRat was 0.903, values higher or lower resulted in shorter detection times.

## Discussion

The number of studies quantifying the appearance of animals to test evolutionary and ecological hypotheses is increasing rapidly with the advancement of imaging methods, computer models and models of animal vision [9–11]. However, the methods developed to determine how conspicuous an animal is against its background have rarely been validated using behavioural data, let alone compared to alternative models. This is an issue that goes beyond simply developing the best techniques to quantify animal appearances; coupling visual models to performance, and determining which metrics are most effective regarding observer behaviour, can also enable predictions about the optimisation of visual signals in nature and in applied tasks. By comparing the performance of a suite of different analysis techniques we have determined the best methods for quantifying detectability from appearance.

We found that there were striking differences between the abilities of different camouflage metrics to predict the capture times of our computer-generated prey. Our study broadly validates the use of the majority of camouflage models used in the literature to date, however there were important differences and exceptions, demonstrating the importance of behavioural validation of these models. The Gabor Edge Disruption Ratio (GabRat) devised for this study performed substantially better than all other metrics; prey with high GabRat values were half as likely to be captured by our human ‘predators’ in a given time-frame than those with low values, demonstrating the potential for powerful evolutionary selection pressures on this metric. Moreover, GabRat was over twice as effective at predicting capture times as the type of algorithm used to generate the prey, and over ten times better than Fourier bandpass, HMAX or luminance difference metrics. Also striking was the relative failure of Canny edge detection-based models (e.g. VisRat and DisRat) to predict capture times when treated as linear predictors (i.e. testing the hypothesis that lower VisRat or DisRat values result in longer capture times). When VisRat and DisRat were fitted non-linearly, the optimal ratios were slightly below one in both cases, where ratios equal to one would fit with a background-matching strategy, and ratios below one are disruptive. The non-linear performance of VisRat and DisRat make them much more difficult to use as predictors of detectability without considerable further investigation of the optimal ratio, which may even change between study systems. The fact that the optimal VisRat and DisRat values were close to one suggests that they are either not measuring edge disruption effectively, or that the optimal level of disruption is very close to a background-matching strategy (which is contradicted by the GabRat result). DisRat was, however, a good predictor of treatment type, able to determine whether prey were generated using the background matching or disruptive algorithms slightly better than GabRat. This demonstrates that the Canny edge methods were not failing due to misidentification of edge artefacts on the prey’s outline. In line with our predictions based on biases in human spatial frequency detection [39], GabRat was most effective with a sigma of 3 pixels. This also suggests that the Canny edge metrics should have been performing optimally for the viewing distance, as they were also calculated at this scale. Taken together this suggests that the angle of the perceived edges relative to the prey’s outline is essential in any model attempting to describe edge disruption, as this is the primary difference between the Canny and GabRat methods that were found to behave so differently in this study. The Canny-edges shown in Fig. 1d demonstrate why basing metrics on the presence of edges alone is not sufficient; the disruptive prey in this example has a large number of detected edges in its outline region that mostly run at right angles to the outline.

The success of GabRat in predicting capture times is all the more striking given the comparatively small area that it measures (e.g. see Fig. 1c). The local comparison zone encompassed approximately 92,000 pixels, and the global camouflage metrics measured 2.1 million pixels. By contrast, the GabRat_σ3_ kernel has a maximum diameter of just 19 pixels, covering an area of approximately 5500 pixels. Even though GabRat only takes into account 0.26% of the available data in the scene, those data were found to be far more effective for predicting capture times than any other data we measured, supporting the notions of Cott [7] and Thayer [15] that the animal’s outline tends to give it away, and suggesting our working definition of edge disruption that takes into account the difference between perceived edges and body outline [8] fits with the observed data. In addition, GabRat is one of the least computationally demanding metrics measured here, and uses biologically inspired methods. The variables required for specifying the model can be based on neurophysiology [20] without the need for guessing variables and thresholds, which sets it apart from the Canny or SIFT-based models [18], or edge-intersecting patch counts [32]. An alternative conclusion is that the pattern and luminance-based metrics we have measured are less effective, perhaps because they fail to model real visual systems adequately, although these methods have a substantial track record in supporting hypotheses in natural systems [16, 22, 40, 41].

In line with Webster et al. [32], we found the Edge-Intersecting Patch Count was a good predictor of capture time, indeed it outperformed all pattern- and luminance-based metrics other than SIFT even though it is blind to the interaction between prey and their backgrounds. However, it is also a less useful metric for generalising to other systems where the edge-intersecting patches are less easily defined. For example, how should discrete patches be distinguished in real prey images, what regions around the prey’s outline should be used, and what visual processing architecture could reproduce these counts? Therefore, although this metric is successful in this study where patches are defined by the prey generation algorithm, we think it an unlikely avenue for fruitful future research into edge disruption compared to metrics that more closely match known neural processing methods.

Contrary to our expectations based on Moreno et al. [42], the best method of quantifying pattern difference was the SIFT model. Although in line with our predictions, prey took longer to capture if they shared more features with their local background region than their global background. This result is similar to experiments demonstrating that captive blue tits *Cyanistes caeruleus* took longer to find prey against backgrounds with higher densities of geometric shapes identical to those on the prey [43]. Our finding suggests that the same effect holds true when natural backgrounds rather than repeated geometric shapes are used. The SIFT model was also the only pattern matching model that performed better than treatment type, which is perhaps surprising given treatment type was blind to the interaction between individual prey and their backgrounds. As predicted, the HMAX models performed better than the Fourier-based bandpass models. The HMAX models that forced comparisons to be made between prey and background without allowing for orientation changes were more effective, for example demonstrating that where there were stripes on the prey these offered the most effective camouflage when they were at the same orientation as the stripes in their background at a similar spatial scale. The Fourier-based global PatternDiff metric performed comparatively well compared to the HMAX metrics even though it is substantially less computationally demanding and less biologically accurate. The other Fourier-based metrics fared less well, although when the global pattern descriptive statistics were combined into an optimal model it predicted capture times well, indeed performing better than any other HMAX or Fourier-based method. However, this model is not directly comparable to the others in that it was adapted to fit the observed data in this study from a large number of degrees of freedom, giving it an unfair advantage. Nevertheless, this process is useful because it highlights those descriptive pattern metrics that best predicted capture times in this study, making it the most useful method for producing information on the importance of different aspects of pattern matching, such as whether spatial scale or energy measures are more important. By contrast the SIFT model provides the least information on what aspects of the general patterns are most important, making it less easy to infer what types of features are most important, how their importance is weighted, and whether these variables and weightings apply equally well to non-human visual systems.

Our data suggest that while matching the average background luminance is important, it is substantially less important than pattern matching or edge disruption metrics. We might have expected to find that pattern and edge disruption should only be important up the point where the prey become so different in average luminance to their background that they stand out (switching from inefficient to efficient search strategies [34]). However, the best luminance predictor (Local Mean Luminance Difference) was dropped from the final model of the best predictors, suggesting that this is not the case. Nor was there autocorrelation between this luminance metric and the best pattern and edge disruption metrics, demonstrating—contrary to our expectation—that prey can mismatch the luminance of their local background and still have a good SIFT pattern match and level of GabRat_σ3_ edge disruption. Prey in real-world situations could have a level of luminance mismatch with their environment beyond those achieved by our computer display, however most background matching prey would not be expected to have such a big luminance difference to their background. The interaction in the final model of best predictors between GabRat_σ3_ and Local SIFT pattern match suggest these metrics can operate in synergy to increase detection times. Although, the effect size of this interaction was small compared to the abilities of GabRat_σ3_ and SIFT to predict capture times on their own.

To our knowledge this study is the first to compare a wide range of different contemporary methods for testing levels of achromatic camouflage. We have validated the use of GabRat_,_ a novel edge disruption metric, while the VisRat and DisRat metrics adopted in the literature to date for investigating edge disruption cannot be used as reliable indicators of detectability. Fourier-based methods were less effective than more complex and biologically plausible methods, they were, however, the most informative for distinguishing different aspects of a pattern’s nature, and were still highly significant predictors of capture time. We would still therefore recommend their use in cases where little is known about the receiver’s visual system. HMAX models, while being the most biologically plausible for quantifying pattern difference were not found to be as effective as SIFT for predicting capture times, indicating that the number of distinct features shared between two patterns is more important than the overall scales, angles and contrast levels. Our use of tree-bark backgrounds photographed under diffuse lighting conditions may also have influenced our findings, and qualitatively different results could be possible against alternative backgrounds, and under different lighting conditions. A number of studies have demonstrated the importance of background complexity in affecting detectability [24, 38, 43], so our findings may not hold against simple backgrounds with low levels of achromatic complexity. Xiao and Cuthill found that feature congestion best predicted human and bird detection of artificial moths [24]. This metric combines local achromatic and chromatic changes, and edge orientation changes. While our study did not consider colour differences or feature congestion explicitly, it measured a number of variables similar to those used in calculating the achromatic components of feature congestion; for example by measuring the number of Canny edges locally, analysing the local pattern energy, and HMAX Gabor filtering, which takes into account edge orientations. While we found that all of these metrics predicted capture times in line with Xiao & Cuthill, they were not as effective as other methods, possibly because they do not consider the prey’s interaction with its background. Future work should compare the effectiveness of these models with images of natural prey, and in wholly natural systems to establish how wide-ranging these findings are to detection times in alternative contexts and with non-human visual systems [24]. In addition, models should be developed that can integrate chromatic cues; experiments on colour discrimination typically involve comparisons between flat colour patches rather than the complex and varied colours encountered in natural search tasks.

## Conclusions

This study demonstrates how best to measure camouflage from appearance, however the same models can also be used to measure signals that stand out from the background [13]. The methods tested in this study are therefore useful for researchers studying the appearance of wide-ranging signals, from sexual and aposematic displays to mimicry and camouflage in fields from evolutionary and sensory ecology to military camouflage and advertising. Model validation in humans can also help to reduce the number of costly animal behaviour experiments required for testing visual hypotheses. Our findings have two main evolutionary implications: first, that we would expect camouflage to be optimised by creating false edges at scales linked to the typical detection distances of the receiver, and second, that while visual systems should have evolved to overcome this weak-spot as effectively as possible, recognising the animal’s outline is still key to detection and/or recognition. Likewise, signals that have evolved to maximise detection (such as sexual or aposematic displays, [13]) should do the opposite, creating coherent edge cues at scales relevant to typical viewing distances.

## Methods

The performance of different models of camouflage measurement was assessed in human touch-screen predation experiments. Although animal vision varies substantially between taxa, human performance in touch-screen experiments has been found to agree with behavioural data from non-humans [27]. Furthermore, spatial visual processing is thought to be similar between taxa [17], suggesting the results based on achromatic human performance should be good indicators of performance in many other vertebrate species.

### Backgrounds and prey generation

Photographs of natural tree bark were used as background images (oak, beech, birch, holly and ash, *n* = 57 images), taken using a Canon 5D MKII with a Nikkor EL 80 mm lens at F/22 to ensure a deep depth of field. Photographs were taken under diffuse lighting conditions. Luminance-based vision in humans is thought to combine both longwave and mediumwave channels [44]. As such we used natural images that measure luminance over a similar range of wavelengths by combining the camera’s linear red and green channels. Next the images were standardised to ensure that they had a similar overall mean luminance and contrast (variance in luminance, see [16]). Images were then cropped and scaled with a 1:1 aspect ratio to the monitor’s resolution of 1920 by 1080 pixels using bilinear interpolation. Images were log-transformed, resulting in a roughly normal distribution of luminance values. A histogram of logged pixel values with 10,000 bins was analysed for each image. The 1st, 50th (median) and 99th percentile luminance values were calculated, and their bins modelled with a quadratic function against the desired values for these percentiles to ensure the median was half way between the luminance at the upper and lower limits. The resulting images all have an approximately equal mean and median luminance, and similar luminance distributions (contrast), and equal numbers of pixels at the upper and lower extremes.

Each prey was generated from the background it was presented against using custom written code similar to that used previously [28]. This methodology creates unique two-tone prey that match the general pattern and luminance of the background (see Fig. 1b). Briefly, for each prey a triangular section of the background image was selected from a random location, 126 pixels wide by 64 pixels high. For disruptive prey the threshold level was calculated that would split the image into the desired proportions (60% background to 40% pattern). For background matching prey a Gaussian gradient was applied to the edges prior to threshold modelling that made it less likely that underlying patterns would come through nearer the edge of the prey. This avoids creating salient internal lines in the background matching prey parallel with the prey’s outline, while ensuring no patterns touch the very edges. If the thresholded proportion was not within 1% of the target limits the process was repeated. Prey were generated with either dark-on-light or light-on-dark patterns, and each participant only received one of these treatments. Dark-on-light prey had the dark value set to equal the 20th percentile of the background levels and the light value set to the 70th percentile. The light-on-dark prey used the 30th and 80th percentiles respectively. The differences between these two treatments are due to the fact that there is slightly more background area than pattern, and these values ensure that the overall perceived luminance of the two treatments is similar to the median background luminance, factoring in the 60/40 split of background to pattern area.

### Calculating camouflage metrics

The camouflage metrics measured in this study fall into seven distinct methodologies, though many of these in turn provide a number of additional variations: Gabor edge disruption ratios (GabRat, first proposed in this study), visual cortex-inspired models based on the HMAX model [20, 45], SIFT feature detection [18, 22], edge-intersecting patch count [32], luminance-based metrics [10, 16], Fourier transform (bandpass) pattern analysis [10, 16, 41], and edge-detection methods to quantify disruption [19, 21, 33]. Where possible, we have used the same terminology for the different metrics as they are used in the literature. Many of these variables can be used to compare a prey target to a specific background region. Therefore, where the metrics allowed, we compared each prey to its entire background image (the ‘global’ region) and to its ‘local’ region, defined as the area of background within a radius of one body-length (126 pixels) of the prey’s outline. The distance of one body length is the largest ‘local’ area that would not exceed the background image limits, because prey were always presented within one body length of the screen edge. This also ensured that the shape of the local region was always uniform, however one body length is also a flexible unit of scale measurement that could be used in other animal systems. Measuring two regions allowed us to test whether a prey’s local or global camouflage matching was more important across the different metrics (see Fig. 1a). If the prey are a very poor luminance match to their backgrounds then we might expect them to stand out enough for the comparatively low acuity peripheral vision to detect them easily using efficient search [34]. However, where the prey are a good luminance and pattern match to their local background they should be most difficult to detect as this would require the participant adopt inefficient search strategies, slowly scanning for the prey. We can make further predictions on the importance of pattern and edge disruption at specific spatial scales given humans are most sensitive to spatial frequencies in the region of around 3–5 cycles per degree [39]. This scale is equivalent to a Gabor filter with a sigma between approximately 2–4 pixels.

For clarity we use the term ‘edge’ to refer to perceived edges based on a given image analysis metric, and ‘outline’ to refer to the boundary between prey and background. Unless otherwise specified, these methods were implemented using custom written code in ImageJ. The GabRat implementation will be made available as part of our free Image Analysis Toolbox [10], code for all other metrics is already available in the toolbox, or is available on request. See Table 1 for an overview of the measurement models.

### Gabor edge disruption ratio (GabRat)

Prey were first converted into binary mask images (i.e. white prey against a black background), a Gabor filter was then applied to each of the pixels around the edge of the prey at a range of angles (four in this study; the Gabor filter settings were identical to those used in the HMAX model below, and Fig. 1e). The angle of the prey’s outline at each point (parallel to the outline) was the angle with the highest absolute energy (*|E|*) measured from the mask image. Each point around the prey’s outline in the original image was then measured with a Gabor filter at an angle parallel to, and orthogonal (at right angles) to the edge at each point. This measured the interaction between the prey and its background. The disruption ratio at each point on the prey’s outline was then calculated as the absolute orthogonal energy (|*E*_*o*_|) divided by the sum of absolute orthogonal and absolute parallel energies (|*E*_*p*_|). Finally, the Gabor edge disruption ratio (GabRat) was taken as the mean of these ratios across the whole prey’s outline:

$$ GabRat=\frac{\Sigma \frac{\left|{E}_o\right|}{\left(\left|{E}_o\right|+\left|{E}_p\right|\right)}}{n} $$

Consequently, higher GabRat values should imply that prey are disruptive against their backgrounds (having a higher ratio of false edges), and lower GabRats imply that the edges of prey are detectable (see Fig. 1c). This process was repeated with different sigma values for the Gabor filter to test for disruption at different spatial frequencies (sigma values of 1, 2, 3, 4, 8 and 16 were modelled in this study). It is therefore possible for prey to be disruptive at one spatial scale or viewing distance, while having more detectable edges at another.

### HMAX models

The HMAX model is biologically inspired, based on an understanding of neural architecture [20]. It breaks down images using banks of Gabor filters [37] that are then condensed using simple steps into visual information for object recognition tasks. It was also found to outperform the SIFT in an object classification comparison [42], so we might therefore expect it to perform best in a camouflage scenario. The HMAX model was developed in an attempt to emulate complex object recognition based on a quantitative understanding of the neural architecture of the ventral stream of the visual cortex [20, 45]. Our HMAX model first applied a battery of Gabor filters to the prey image, and the local and global background regions. The Gabor filters were applied at four angles and ten different scales, with Gamma = 1, phase = 2π, frequency of sinusoidal component = 4, minimum sigma = 2, maximum sigma = 20, increasing in steps of 2. C1 layers were created following Serre et al. [20] by taking the maximum values over local position (with a radius of sigma + 4) and the neighbouring scale band. The mean of each scale band in the prey’s C1 was then calculated as we wished to compare the prey’s overall pattern match rather than a perfect template match, which would test a masquerade rather than background-matching hypothesis [6]. This C1 template was then compared to each point in the local surround and entire (global) background image. The average match between the prey’s C1 layer and the C1 layers of each region was saved along with the value of the best match and the standard deviation in match (a measure of heterogeneity in background pattern matching). This model was run both with and without an allowance for the template to rotate at each point of comparison. When rotation was allowed the angle that had the best match at each comparison site was selected. The HMAX model with rotation describes how well the moth’s average pattern (i.e. angles, scales and intensities) matches the background if the prey can rotate to the optimal angle at each point. The model without rotation forces the prey to be compared to its background at the same angles, so should be a better predictor in this study where the prey’s angle relative to its background is fixed.

### SIFT feature detection

Scale Invariant Feature Transform (SIFT, [18]) models were primarily developed for object recognition and rapidly stitching together images by finding sets of shared features between them even if they occur at different scales or angles. Although the SIFT models share some similarities with known biological image processing and object recognition at certain stages, such as the inferior temporal cortex [46], the method as a whole is not intended to be biologically inspired, although it has been applied to questions of animal coloration [22].

The SIFT function in Fiji (version 2.0.0 [47]) was used to extract the number of feature correspondences between each prey and its local and global background without attempting to search for an overall template match. Settings were selected that resulted in a large enough number of correspondences that the count data would exhibit a normal rather than Poisson distribution, but that was not too slow to process. These settings produced roughly 300 features in the prey, and 300,000 in the background in a sub-sample run to test the settings. The initial Gaussian blur was 1, with 8 steps per scale octave, a feature descriptor size of 4, 8 orientation bins and a closest to next-closest ratio of 0.96. Prey and their local background regions were measured in their entirety against a white background. As it stands this methodology might not therefore be suitable for comparing prey of different shapes and sizes without further modification and testing.

### Edge-intersecting cluster count

The number of cases where patterns intersected the preys outline (following [32]) were summed in each prey using a custom written script. Background matching prey had no instances of edge intersections, which would create zero inflation and violate model assumptions. We therefore analysed a subset of the data containing only disruptive prey for testing this metric.

### Luminance-based metrics

Prey were compared to their local and global background regions using a number of luminance-based metrics that could affect capture times. Luminance was taken to be pixel intensity values. LuminanceDiff was calculated as described in Troscianko et al. [10], as the sum of absolute differences in the counts of pixel numbers across 20 intensity bins, essentially the difference in image luminance histograms. This measure is suitable where the luminance values do not fit a normal distribution, which is the case with our two-tone prey. Mean luminance difference was the absolute difference in mean luminance values between prey and background regions. Contrast difference was calculated as the absolute difference in the standard deviation in luminance values between prey and background region. Mean local luminance was simply the mean pixel level of the local region. This was not calculated for the entire background image because they had been normalised to have the same mean luminance values.

### Bandpass pattern metrics

Fourier Transform (bandpass) approaches [17, 40] only loosely approximate the way visual systems split an image into a number of spatial frequencies, however, they have a long and successful track record of use in biological systems, are fast to calculate and provide output that can be used flexibly to test different hypotheses [16, 41]. Fast-Fourier bandpass energy spectra were calculated for the prey and their local and global background regions using 13 scale bands, increasing from 2px in multiples of √2 to a maximum of 128px [10]. PatternDiff values were calculated as the sum of absolute differences between energy spectra at each spatial band [10]. This metric describes how similar any two patterns are in their overall level of contrast at each spatial scale. Descriptive statistics from the pattern energy spectra were also calculated, these being: maximum energy, dominant spatial frequency (the spatial frequency with the maximum energy), proportion power (the maximum energy divided by the sum across all spatial frequencies, mean energy and energy variance (the standard deviation in pattern energy, a measure of heterogeneity across spatial scales) [10, 41]. A metric similar to the multidimensional phenotypic space used by Spottiswoode and Stevens [48] was calculated from these descriptive statistics. However, rather than sum the means of each descriptive pattern statistic, a Euclidean distance was calculated after normalising the variables so that each had a mean of zero and standard deviation of one (ensuring equal weighting between pattern statistics). We termed this metric ‘Euclidean Pattern Distance’. In addition, a full linear mixed model was specified containing all descriptive pattern statistics and all two-way interactions between them (the model was specified in the same form as other linear mixed models in this study, see statistical methods below). The full model was then simplified based on AIC model selection.

### Canny edge detection methods

Canny edge detection methods were applied following Lovell et al. [21] and Kang et al. [33], using a Java implementation of the method [49]. The Canny edge filter was applied to each image with the settings specified by Lovell et al., using a sigma of 3 and a lower threshold of 0.2. The upper threshold required by the Canny edge detection algorithm was not specified by Lovell et al., so a value of 0.5 was selected that ensured there would be no bounding of the data where no edges were detected. Following Lovell et al., the prey’s outline region was specified as being four pixels inside the prey’s outline and 8 pixels outside (see Fig. 1d). As above, two background regions were measured; local and global, although the 8px band around the prey’s outline was not included in the local or global regions. We measured the mean number of Canny edge contours in each region (i.e. the number of edge contour pixels in each region divided by the total number of pixels in each region to control for the differences in area being measured). It is unclear whether Lovell et al. applied this control, however given the areas being measured are fixed in this experiment (all prey and backgrounds are the same size) this would not affect the overall outcome. VisRat was calculated as the mean Canny edge contours found in the background region (either local or global) divided by the mean Canny edge contours found in the prey’s outline region (termed ContEdge by Kang et al.). DisRat was calculated following Kang et al. as being the mean Canny edge contours found inside the prey (termed MothEdge by Kang et al.) divided by ContEdge. Both VisRat and DisRat required a log transformation to demonstrate a normal error distribution.

### Experimental setup

Prey were presented at a random location against their background using custom written HTML5/Javascript code on an Acer T272HL LCD touch-screen monitor. The display area was 600 mm by 338 mm, 1920 by 1080 pixels. The monitor’s maximum brightness was 136.2 lux, and minimum was 0.1 lux, measured using a Jeti Specbos 1211 spectroradiometer. The monitor’s output fitted a standard Gamma curve where brightness (lux) = 8.362E-4*(x + 25.41)^2.127*exp (−(x + 25.41)/3.840E11), where x is an 8-bit pixel value. The monitor was positioned in rooms with standard indoor lighting levels and minimal natural light. Prey were 39.38 mm wide (126 pixels, approximately 4.59°) by 20.03 mm (64 pixels, approx. 2.30°) high, viewed from a distance of approximately 500 mm (approx. 27.9 pixels per degree). If participants touched the screen within the bounds of the prey the code recorded a capture event to the nearest millisecond, a high-pitched auditory cue sounded, and a green circle appeared around the prey for 1 s. If they touched the screen outside the prey’s bounds a low-pitched auditory cue sounded, and they were not progressed to the next screen. If the participant failed to find the prey after 20 s (timeout) a red circle appeared around the moth for 1 s and a low-pitched auditory cue sounded, and capture time was set at 20 s (this occurred in just 3.5% of slides). In addition, for every successful or failed capture event, or timeout event the location of the touch was recorded. Participants started each session by clicking a box asking them to ‘find the artificial triangular “moths” as fast as possible’, confirming that they were free to leave at any point, and that it should take less than 10 min to complete (all trails were under 10 min). A total of 120 participants were tested, each receiving 32 slides (i.e. 32 potential capture events), creating a total of 3840 unique prey presentations.

### Statistics

All statistics were performed in R version 3.2.2 [50]. For each camouflage metric a linear mixed effects model was specified using lme4 (version 1.1-10). The dependent variable in each model was log capture time. The main aim of this study was to establish which camouflage measurement models best predicted human performance, and as such we compared the variance in capture times explained between models. The multiple models created here increase the likelihood of making a type I error, however, alpha correction methods (such as Bonferroni or Šidák corrections) are not strictly suitable for these data as many of the models are non-independent, measuring subtly different versions of the same effect, and they would increase the likelihood of type II errors. As such we focused on the level of variance explained by each variable and its associated effect sizes for ranking the models. A number of variables known to affect capture times were included in the model to reduce the residual variance to be explained by the camouflage metrics [28]. These were the X and Y screen coordinates of the prey, each included with quadratic functions and with an interaction between them to reflect the fact that prey in the centre of the screen were detected sooner than those at the edges or corners. A variable was used to distinguish the first slide from all subsequent slides, describing whether the participant was naive to the appearance of the prey. Slide number was fitted to account for learning effects. Random effects fitted to the model were participant ID and background image ID, allowing the model to ignore the differences in capture time between participants or against specific backgrounds when calculating the fixed effects. Each camouflage metric was substituted into this model and the deviance explained by each camouflage metric was calculated using the pamer function of LMERConvenience-Functions (version 2.10). All camouflage metrics were continuous variables transformed where necessary to exhibit a normal error distribution with the exception of treatment type, which was categorical (background matching or disruptive prey). An additional final model was assembled based on the best performing edge disruption metric, pattern matching metric and luminance matching metric with two-way interactions between them. These variables were checked for autocorrelation using Spearman covariance matrices [51]. This full model was them simplified based on AIC maximum likelihood model selection to determine whether the best camouflage predictors interact in synergy to better predict camouflage than any single metric on its own.

## Additional file

Additional file 1: Table S1. Model terms in the simplified model of bandpass-based descriptive statistics. Prey X and Y screen coordinates are added with polynomial fits and an interaction. (DOC 140 kb)

## Abbreviations

HMAX: “Hierarchical Model and X”
SIFT: “Scale invariant feature transform”
