# Supplementary material for: Quantifying camouflage: how to predict detectability from appearance
Source: BMC Evol Biol. 2017 Jan 6;17:7. doi: 10.1186/s12862-016-0854-2 (PMC5217226; doi:10.1186/s12862-016-0854-2)

*Additional file 1: Table S1.* Model terms in the simplified model of bandpass-based descriptive statistics. Prey X and Y screen coordinates are added with polynomial fits and an interaction.


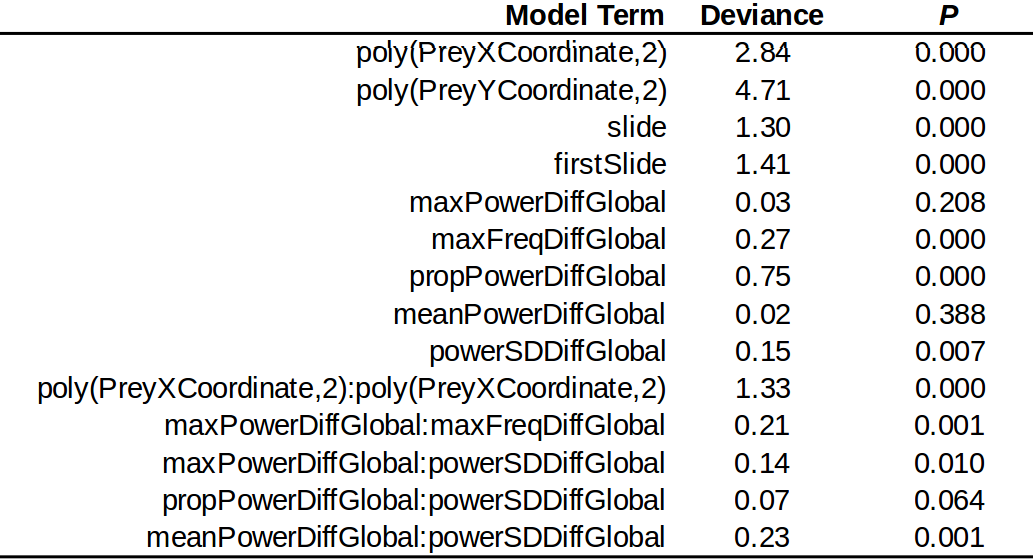

Supplement: Additional file 1: Table S1. — Model terms in the simplified model of bandpass-based descriptive statistics. Prey X and Y screen coordinates are added with polynomial fits and an interaction. (DOC 140 kb) [file 12862_2016_854_MOESM1_ESM.doc]
